# Filling the gaps sustainably: an island case study of rural dental workforce challenges

**DOI:** 10.3389/fdmed.2025.1640643

**Published:** 2025-09-17

**Authors:** Silvana Bettiol, Arish Naresh, Shahrukh Khan

**Affiliations:** ^1^ College of Health and Medicine, University of Tasmania, Hobart, Tasmania; ^2^ Department of Community Oral Health Services, Health New Zealand Tairāwhiti, Gisborne, New Zealand

**Keywords:** sustainable development goals, oral health, workforce distribution, access to care, islands

## Abstract

Tasmania faces ongoing challenges in providing equitable oral health care across its geographically dispersed and ageing population. Tasmania has the lowest dental practitioner-to-population ratio in Australia, alongside long wait times and limited access to care in rural and regional areas. This perspective explores the demographic and workforce distribution trends that influence oral health service delivery in Tasmania using Census data, regulatory records, and key public sector audits. We highlight critical gaps in service provision and policy, reflect on the implications of the workforce maldistribution, argue for expanding the oral health therapy workforce and explore the transdisciplinary approaches currently implemented that prioritise sustainable, community-integrated oral health care. Such models align with the United Nations Sustainable Development Goals (SDGs), particularly SDG 3 (Good Health and Well-being), but also encompass SDGs 4, 5, 6, 8, 9, 10, 13 and 17, which respond to environmental and social contexts. This perspective highlights the intersections of planetary health, oral health, and community well-being, arguing that sustainable, health-centred solutions must address both human and environmental health outcomes.

## Introduction

The island of Tasmania is Australia's smallest state, renowned for its clean air, natural beauty, and high quality of life. A 2025 study by Monash University found that Tasmania ranked as the healthiest state in Australia based on its Environmental Quality Health Index (EQHI), with Hobart named the healthiest capital city (1, 2), This index highlighted the state's favourable air quality, access to green space, and socio-environmental conditions, reinforcing Tasmania's image as an ideal place to live (2). Despite these positive environmental attributes, Tasmania faces persistent health challenges, particularly in accessing timely and equitable healthcare services.

More than half of all Tasmanian adults (58.4%) live with at least one chronic condition the highest proportion of all jurisdictions in Australia (3). The most prevalent chronic conditions include musculoskeletal disorders, cancer, mental health issues, cardiovascular disease, and diabetes (4). Life expectancy in Tasmania is lower than the national average (5). The population's poor health is linked to sociodemographic factors such as its older population, lower education attainment, lower income levels, and more people living in rural areas than elsewhere in Australia (2, 6). Nevertheless, many chronic conditions are preventable or manageable through early detection and coordinated primary care interventions (7).

Tasmania has the highest rural and remote population dispersion in Australia, with 32% of young people residing outside the four major local government areas (8). The healthcare system is a complex mix of services and programs delivered by a range of providers, often creating confusion for patients navigating care (6). Tasmania's rising proportion of residents aged 65 and older, will significantly increase demand for aged care and health services. This trend reinforces the need for a sustainable, integrated health system aligned with SDG 3 (Good Health and Well-being), which promotes equitable access to essential healthcare (9). In addition, Tasmania faces ongoing challenges in recruiting and retaining healthcare professionals. Labour market data indicate a persistent shortage of skilled health workers (10), compounded by limited training opportunities for allied health professionals compared to mainland states (11). These challenges are most pronounced in regional and rural communities, where workforce shortages and service gaps disproportionately affect vulnerable populations including in oral health.

In response to global concerns, the World Health Organization (WHO) and the FDI World Dental Federation have prioritised oral health within broader public health agendas. The FDI Vision 2030 reaffirms the importance of oral health in managing noncommunicable diseases and achieving health equity (12, 13). More recently, FDI has advocated for integrating oral health into electronic health records to support more holistic and system-wide care (14).

While no specific United Nations Sustainable Development Goal (SDG) target explicitly addresses oral health, oral health professionals are recognised as part of the broader health workforce under SDG3: Target 3.c.1 (15). Following years of advocacy, the World Health Assembly adopted Resolution WHA74.5 on oral health, which set a renewed global policy agenda. The Global Strategy and Action Plan on Oral Health (2023–2030) outlines goals aligned with SDG 3 and formally positions oral health within the global health discourse (16, 17). Oral health crisis can be viewed as part of a broader, global challenge to ensure the provision of equitable, accessible, and sustainable healthcare systems that contribute to planetary health, as emphasised by SDG 13 (Climate Action) and the emerging field of planetary health, which links human health with environmental sustainability (18).

This paper explores the intersection of the Sustainable Development Goals and oral health in Tasmania, focusing on workforce distribution and access to care.

## A snapshot of the oral health situation in Tasmania

Poor oral health is associated with being older, low levels of education and income, being Indigenous, and with living in rural areas (19–21). These five sociodemographic factors negatively influence oral health more in Tasmania than in mainland Australia compounded by structural challenges such as rural workforce shortages and long public dental waiting lists. COVID-19 intensified these issues by pausing non-urgent care, (22–24). Similar challenges have been documented in other regions such as New Zealand, Canada, Scotland's Western Isles and Hawaii, where rural and remote populations face workforce shortages limited preventive care, and inequitable service distribution (25–28). Comparisons with these regions suggest that integrated service models, mobile clinics, community engagement and expanded roles for oral health therapists can improve access and outcomes, offering insights for potential policy adaptations in Tasmania.

Yet, Tasmania has much to commend. Oral Health Services Tasmania (OHST), the island's public oral health sector, operates 33 clinics statewide, including three Special Care Dental Units in hospitals (29). Its 350-strong multidisciplinary team offers emergency, general care to all children under 18 years of age. Children aged 0–17 can access free care in either the private or public sectors under the Commonwealth-funded Child Dental Benefits Schedule. OHST offers general and denture care at subsidised rates to eligible adults holding Commonwealth Health Care Cards, which are allocated based on a person's income and family status (30, 31).

OHST's innovative care includes graduate training programs (32), digital denture technology, and the expanded use of digital orthopantographic X-radiographs (33). This technology-enabled and team-based model supports SDG 9 (Industry, Innovation and Infrastructure) and workforce development. Partnerships with external providers like the Royal Flying Doctor Service Tasmania (34) and with some private practitioners demonstrate SDG 17 (Partnerships for the Goals) by leveraging collaboration and access in underserved areas (24).

Preventive programs for schoolchildren (e.g., Fissure Sealants, Fluoride Varnish, Lift the Lip) and pregnant women (Smiles for Two) reflect SDG 10 (Reduced Inequalities; Target 10.2) and SDG 3 (Target 3.8), while enhancing community health literacy in line with SDG 4 (Quality Education). Special services, including Oral and Maxillofacial Surgery Tasmania offer conscious sedation for medically complex or hospitalised patients (24).

Nearly all (96.6%) Tasmanians with access to reticulated water receive fluoridated water (35–37). This plays a critical role in preventing dental caries and in promoting oral health equity (38), particularly in communities with limited access to dental services and support SDG 6 (Clean Water and Sanitation; Target 6.1).

Conversely, about 13% of Tasmanians smoke daily (39), with higher rates in vulnerable groups including pregnant women (40). Vaping among youth is rising, with one in five having vaped and reports of illegal sales within schools (41). Smoking is strongly associated with periodontal (gum) disease, tooth loss, oral mucosal lesions, and a leading cause of oral cancer. It can contribute to delayed healing after dental procedures and reduces the success of treatments such as implants and periodontal therapy. From October 2024, e-cigarettes will be prescription-only in Tasmania, a legislation aligned with SDG 3 (Target 3.a).

Systemic barriers, including a reactive funding model, under-served regions, and limited integration of prevention undermine population access to routine care. Aged care residents and incarcerated individuals are disproportionately affected (24). Collaborative programs led by the OHST, Education Department, the Australian Dental Association, and private insurance providers attempt to improve affordability, reach, and prevention across the state (42, 43).

## Population dynamics and workforce distribution

Tasmania's estimated population was 575,496 in mid-2024, growing slowly at 0.31% annually (44). The population dynamics are influenced by net interstate migration, overseas migration and natural increase (45). Looking ahead, projections estimate that the population will reach approximately 641,045 by 2053 (46) and expected to age rapidly over the next 30 years (47). Over 5% (5.4%, 30,000 people) of Tasmanians identified as Aboriginal and/or Torres Strait Islander in 2021, making up 5.4% of the state's population (48). The largest Indigenous populations were in the cities of Launceston, Glenorchy, and Clarence, while Circular Head and Flinders Island have the highest proportions relative to their local populations (49).

These demographic trends suggest that demand for health services, including dental care, will continue to rise, particularly among older adults. Yet this growth has not been matched by proportional increases in the oral health workforce, especially outside of metropolitan areas.

## Professional dental workforce

According to the National Rural Health Alliance, Tasmania has the lowest dental practitioner density in Australia, with only 26.5 dental practitioners per 100,000 residents which is far below the national average of 72.5 per 100,000 (50). Most dentists (82.1%) across Australia practise in metropolitan areas, and only 0.1% serve in very remote regions, reflecting stark urban–rural disparities that are particularly evident in Tasmania (51).

In June 2023, there were 19,179 registered dentists nationally, with the women entering the profession increasing to 52.4% up from 43.5% in 2018/19 (52). However, the proportion of younger dentists (under 35 years) declined slightly to 29.6%. The Australian Capital Territory (ACT) recorded the highest dentist-to-population ratio (79.4), while Tasmania (49.6) and the Northern Territory (46.9) were the lowest. These figures underscore Tasmania's enduring workforce gap (53).

In contrast, dental hygienists, therapists, and oral health therapists (5,314 practitioners) make up 10.8% of the dental workforce and 0.6% of the total regulated health workforce with stronger rural presence including 7.8% practising in regional areas and 7.3% in large rural towns (54). The practising density stood at 20.1 per 100,000 population. This workforce was predominantly female (91.3%). While the average age was 43.5 years. Age distribution showed 44.8% under 35 years, 40.9% aged 35–54, and 14.3% over 55, although 79.2% continued to work in metropolitan areas (55).

Practitioner density remained low in Tasmania, whereas South Australia (39.7) and Western Australia (29.1) showed comparatively higher ratios (AHPRA, 2024). Encouragingly, this group has grown by 14.5% over the past five years, with the greatest gains in large rural and inner regional areas, aligning with SDG 3: Target 3.c, which calls for investment in and equitable distribution of the health workforce (56).

By December 2024, the total number of registered dental practitioners in Australia rose to 28,677, comprising 25,454 with general registration, 1,882 with both general and specialist registration, 74 with specialist registration, 59 with limited registration, and 1,205 with non-practising registration (57). Of these, 159 practitioners (0.6%) identified as Aboriginal and/or Torres Strait Islander, a figure unchanged from the previous year, underscoring the need to strengthen Indigenous representation in oral health professions (58).

Meanwhile, the dental services industry generated $10.9 billion in revenue in 2022–23, employing 56,810 jobs across 19,119 businesses (58). However, sectoral growth was marginal at just 0.1% over five years, constrained by inflationary pressures and increasing fragmentation as well as disruption by COVID19. Looking ahead, annual growth is projected at +2.0%, reaching $12 billion by 2028 (58).

Most dental services are privately funded, with $2.9 billion in private health insurance benefits paid for 44.8 million services in 2021–22 (58). For context, the broader health services industry generated $202.4 billion in revenue in 2023–24, with an 11.4% profit margin and nearly 1 million workers. Dentists, hospitals, GPs, and specialists remain major contributors (59).

Despite trends toward corporatisation of practices, access to dental care in regional Tasmania remains limited due to a thin and uneven workforce, infrastructure constraints, and restricted public funding. These factors collectively pose a challenge to achieving equitable oral health outcomes and underscore the urgency of targeted workforce development initiatives in underserved regions.

## Workforce scope and strategic use of Oral Health Therapists (OHTs) in Tasmania

Although limited empirical evidence links the Sustainable Development Goals (SDGs) directly to dental workforce distribution, they provide a strategic framework to guide policy and investment decisions. In Tasmania, where access disparities persist, aligning workforce development with the SDGs offers a promising path forward.

The oral health workforce in Tasmania includes several regulated professional categories under the Dental Board of Australia (DBA) (60), each with distinct scopes of practice. *“Oral health therapists have qualifications in both dental therapy and dental hygiene. They can assess, diagnose, treat, manage, and prevent oral disease. Their scope includes restorative procedures, fillings, extractions, periodontal care, and broader oral health promotion across all age groups.”*

Evidence from international contexts shows that dental therapists and oral health therapists (OHTs) can improve oral health access and outcomes in underserved populations. For example, deployment of dental therapists in Alaska Native communities has been linked to increased preventive care, improved access, and reductions in extractions and emergency dental visits (61). Similarly, in the United States, dental therapists are recognised as new oral health practitioners who expand workforce capacity and reduce disparities in access for underserved populations (62).

In Tasmania, OHTs are well-suited for workforce deployment models such as those implemented by Oral Health Services Tasmania (OHST), where they are more commonly employed in underserved areas than dentists. This strategy aligns with SDG3: Target 3.c, which promotes increased health workforce investment and more equitable distribution of healthcare providers. The process advances SDG 10 (Reduced Inequalities) by addressing persistent gaps in access for rural and remote populations. The deployment of OHTs in rural areas also supports SDG 11 (Sustainable Cities and Communities) by strengthening the health resilience of these communities.

Workforce sustainability of OHTs is concerning with national average age of OHTs at 43.5 years, with Tasmania showing a higher proportion of practitioners aged 50 and over (56). The profession is predominantly female (over 90%), raising gender-related considerations regarding career longevity, retention, and progression. These issues are relevant to SDG 5 (Gender Equality), Target 5.5, which calls for full and effective participation of women in leadership and decision-making roles. The need for continued investment in training pathways and continuous professional development is essential, aligning with SDG 4 (Quality Education) and SDG 8 (Decent Work and Economic Growth), particularly Target 8.5.

## Aboriginal and torres strait islander dental workforce

The National Health Workforce Dataset (NHWDS) provides insights into the low but growing representation of Aboriginal and Torres Strait Islander professionals in Australia's health workforce (63). Between 2015 and 2019, the number of registered Aboriginal and Torres Strait Islander allied health practitioners nearly doubled from 688 to 1,354, and Health Practitioners increased from 322 to 584 (64, 65). As of March 2025, 159 registered dental practitioners in Australia identify as Aboriginal and/or Torres Strait Islander, accounting for only 0.6% of the profession.

Although representation remains low, initiatives show the potential of Aboriginal and Torres Strait Islander workforce participation to deliver culturally safe care. Aboriginal dental assistants have been trained to safely apply fluoride varnish in regional, rural, and remote schools, improving preventive care and access for children (66). Likewise, Aboriginal health staff in maternal oral health emphasise the importance of trust, cultural understanding, and continuity in supporting Aboriginal women, underscoring the value of Indigenous workforce participation in improving outcomes (67).

These developments support SDG 3 and SDG 10 by improving access and equity, while also advancing SDG 4 (Quality Education) through strengthened training pathways. Efforts to strengthen Indigenous representation reflect the importance of cultural safety (68, 69) and align with SDG 16 (Peace, Justice and Strong Institutions), which promotes inclusive, equitable systems.

## Why are dental practitioners not going rural?

A rural background and rural training are strong predictors of rural practice. Graduates from rural areas are significantly more likely to return to these regions (70–72). Targeted recruitment and training programs not only help alleviate workforce shortages but also foster cultural competence in care delivery. Despite this, long-term retention remains a challenge. University-led outreach programs play a part to the solution, exposing students to rural practice early (73, 74), while CPD requirements help maintain care quality (75).

While some practitioners value the rural lifestyle and community connection, financial and lifestyle incentives continue to favour urban practice (76). Common barriers for health professionals in general include professional isolation, limited career progression, and reduced access to continuing professional development (77). Policy mechanisms such as student loan support, relocation support, and tax incentives are inconsistently applied and often inadequate or predictors of eventual rural practice (78, 79).

Ongoing revision and renewal of national strategies for rural dental training is needed to improve access and workforce distribution (80). No single solution is adequate addressing this issue requires a systems approach that aligns education, incentives, and rural infrastructure support.

Expanding and retaining the rural dental workforce directly supports SDG 8 (Decent Work and Economic Growth). Strengthening rural health workforce contributes to local economies, improves access to essential services, and supports sustainable development. It also reinforces broader goals around equity, resilience, and sustainable community development.

## Policy and systems implications

Tasmania presents a strong case for why more empirical evidence is needed to evaluate progress towards the key SDGs identified in this perspective on oral health. Despite promising policy shifts and workforce innovations, it remains unclear to what extent current strategies are advancing these global goals in practice over time. Research is urgently required to assess real-world outcomes, track progress against equity and sustainability indicators, and inform future investment. Guided by the National Rural Health Alliance (NRHA) (50) and other stakeholders, rethinking dental workforce training and retention is critical.

We recommend more actionable, specific policy measures including; expanding rural training pathways and recruiting students from rural backgrounds to address maldistribution; providing early, practical rural placements to foster long-term retention and improve cultural competence in care delivery; aligning dental policy with universal health coverage principles to ensure to ensure equitable, lifelong access; standardising and strengthening financial, relocation, and career progression incentives to make rural practice more attractive; and guaranteeing access to continuing professional development and mentorship opportunities.

Embedding oral health in interprofessional education and integrating dental services within primary health teams, particularly in community settings can strengthen care as seen across multiple settings. Flexible, community-based models like the Primary Care Rural Integrated Multidisciplinary Health Services (PRIM-HS) offer more sustainable solutions (81) supported by a funding shift from emergency-focused treatment to preventive care.

Future policy directions must prioritise sustained funding for research, implementation, and evaluation of community-integrated oral health programs to ensure evidence-based approaches are scalable, equitable, and responsive to Tasmania's changing demographic and environmental contexts.

## Conclusion

Tasmania's oral health system faces ongoing challenges: workforce shortages, service inequities regional disparities, and rising preventable disease. Innovative partnerships such as those with the Royal Flying Doctor Service and public–private collaborations may alleviate some pressures. However, gaps remain, particularly in preventive care and equitable access.

This perspective underscores the relevance of nine Sustainable Development Goals (SDGs) in guiding equitable and sustainable oral health reform in Tasmania. By aligning with global priorities, particularly around prevention, education, workforce development, infrastructure, climate resilience, and partnerships, islands can build a more inclusive and effective oral health system. Realising this vision will require ongoing collaboration, policy innovation, and a commitment to data-driven action to ensure that no community is left behind.

## Data Availability

The original contributions presented in the study are included in the article/Supplementary Material, further inquiries can be directed to the corresponding author.
